# ppb-Level Selective Hydrogen Gas Detection of Pd-Functionalized In_2_O_3_-Loaded ZnO Nanofiber Gas Sensors

**DOI:** 10.3390/s19194276

**Published:** 2019-10-02

**Authors:** Jae-Hyoung Lee, Jae-Hun Kim, Jin-Young Kim, Ali Mirzaei, Hyoun Woo Kim, Sang Sub Kim

**Affiliations:** 1 Department of Materials Science and Engineering, Inha University, Incheon 22212, Korea; 22171057@inha.edu (J.-H.L.); kjhhb5331@hanyang.ac.kr (J.-H.K.); 22171286@inha.edu (J.-Y.K.); 2 The Research Institute of Industrial Science, Hanyang University, Seoul 04763, Korea; alisonmirzaee@yahoo.com (A.M.); hyounwoo@hanyang.ac.kr (H.W.K.); 3 Department of Materials Science and Engineering, Shiraz University of Technology, Shiraz 71557-13876, Iran; 4 Division of Materials Science and Engineering, Hanyang University, Seoul 04763, Korea

**Keywords:** Pd, In_2_O_3_, ZnO, hydrogen gas, gas sensor, sensing mechanism

## Abstract

Pd nanoparticle-functionalized, xIn_2_O_3_ (x = 0.05, 0.1, and 0.15)-loaded ZnO nanofibers were synthesized by an electrospinning and ultraviolet (UV) irradiation method and assessed for their hydrogen gas sensing properties. Morphological and chemical analyses revealed the desired morphology and chemical composition of the synthesized nanofibers. The optimal gas sensor namely Pd-functionalized, 0.1In_2_O_3_-loaded ZnO nanofibers showed a very strong response to 172–50 ppb hydrogen gas at 350 °C, which is regarded as the optimal sensing temperature. Furthermore, the gas sensors showed excellent selectivity to hydrogen gas due to the much lower response to CO and NO_2_ gases. The enhanced gas response was attributed to the excellent catalytic activity of Pd to hydrogen gas, and the formation of Pd/ZnO and In_2_O_3_/ZnO heterojunctions, ZnO–ZnO homojunction, as well as the formation of PdH_x_. Overall, highly sensitive and selective hydrogen gas sensors can be produced based on a simple methodology using a synergistic effect from Pd functionalization and In_2_O_3_ loading in ZnO nanofibers.

## 1. Introduction

Hydrogen (H_2_) gas is a green, renewable, and sustainable energy source [1]. The main advantage of H_2_ is its abundance and its final combustion product being H_2_O, which is again a source of H_2_ [2]. On the other hand, despite the high practicality of H_2_, its flammability over a wide range of concentrations (4%–75%) and an auto-ignition temperature of 250–400 °C makes it extremely dangerous [3]. Furthermore, it is a colorless, odorless, and tasteless gas that cannot be detected by human senses [4]. Accordingly, during storage, transport and use, an unexpected leakage of hydrogen can be catastrophic. As the use of hydrogen is becoming increasingly commonplace in different industries, the early detection of hydrogen gas using gas sensors is of prime importance [5].

Chemiresistor-based gas sensors are one of the most popular types of sensors for hydrogen gas detection [6]. Among them, those based on ZnO are often reported for the sensing of hydrogen gas [7,8,9]. Indeed, ZnO, with n-type conductivity, has some advantages for use as a hydrogen sensing layer. ZnO has a wide band gap of 3.37 eV, high mobility of charge carriers, high thermochemical stability, and low production cost [10]. On the other hand, to enhance the hydrogen sensing properties of ZnO-based gas sensors, including their response to hydrogen gas and selectivity, different strategies, such as the generation of n–n [11] or n–p [12] heterojunctions, noble metal decoration [13,14], and morphology engineering [15], have been reported.

In_2_O_3_ is an n-type metal oxide with a wide band gap of 3.55–3.75 eV [16], which has been reported to have good sensing properties toward a range of gases, such as acetaldehyde [17], CO/NO_2_ gases [18], and glycol vapor [19]. Accordingly, it appears that heterojunctions of ZnO with In_2_O_3_ have good sensing performance to toxic gases. For example, ZnO–In_2_O_3_ nanocomposites have been reported for the detection of n-butanol gas [20,21], trimethylamine [22], ammonia [23], acetone [24], ethanol [25], and NO_2_ gas [26]. An enhanced gas response is generally attributed to modulation of the gas sensor resistance in heterojunctions between In_2_O_3_ and ZnO, which have different work functions.

It is well known that the morphology of sensor materials greatly affects the sensor performance [27,28,29]. Electrospinning is a facile, low-cost, and high yield technique for fabrication of various oxides with fibrous morphology in nanoscale so-called nanofibers (NFs) which are suitable for gas sensing studies [30]. For example, V_2_O_5_-doped TiO_2_/WO_3_ ceramic structures were prepared by electrospinning technique for humidity sensing studies [31]. In another work, TiO_2_/WO_3_ heterogeneous structures were produced by electrospinning and subsequent sintering for sensing studies [32].

The main objective of this work was the systematic study of effects of different In_2_O_3_ loading on the ZnO NFs for the detection of very low concentrations of hydrogen gas. Since hydrogen is a reducing gas, at high sensing temperature it is expected that as-formed n-In_2_O_3_/n-ZnO heterojunctions are greatly affected by hydrogen gas. In addition, to further enhance the sensing properties, Pd nanoparticles (NPs) were functionalized on In_2_O_3_-loaded ZnO NFs. Promising effects of Pd to hydrogen gas are already well known [33]. Pd not only dissociates oxygen gas and hydrogen gas easily on the surface of the sensing layer but can also be converted to PdH_x_ [34], resulting in the gas sensor showing a greater change in resistance.

In this study, Pd NP-functionalized xIn_2_O_3_ (x = 0.05, 0.1, and 0.15)-loaded ZnO NFs were synthesized using an electrospinning and ultraviolet (UV) irradiation method, and their hydrogen gas sensing properties were investigated. The optimal In_2_O_3_ loading and amount of Pd NPs led to a very strong response to ppb levels of hydrogen gas in a selective manner. Ultrahigh sensitive hydrogen gas sensors can be realized using this methodology and design.

## 2. Experimental

### 2.1. Preparation of the Solution for Electrospinning

The synthesis of a viscous solution for the preparation of In_2_O_3_-loaded ZnO NFs was based on previous papers [35,36]. Analytical grades of polyvinyl alcohol (PVA, MW = 80,000), zinc chloride dihydrate (ZnCl_2_·2H_2_O), and indium nitrate hydrate (In(NO_3_)_3_·4.5H_2_O) were provided by Sigma-Aldrich (St. Louis, MO, USA). Deionized (DI) water was used as a solvent for the preparation of all solutions. First, PVA was added to DI water and stirred (400 rpm) at 70 °C for 4 h to produce a 10 wt.% PVA aqueous solution. Subsequently, 1 g of ZnCl_2_·2H_2_O and calculated amounts (0.05–0.15 molar ratio) of In(NO_3_)_3_·4.5H_2_O were added to the polymer solution and stirred (400 rpm) for 12 h at 70 °C. The final viscous solutions (280 mPa.s) for electrospinning were then prepared. The amount of In precursor was changed to prepare solutions with different nominal compositions of In_2_O_3_ (0.05, 0.1, and 0.15 molar ratio) for the final products.

### 2.2. Electrospinning Process

The electrospinning solution was loaded into a plastic syringe with a metallic needle with an inner diameter of 0.021 mm. To initiate and accelerate electrospinning, a large positive voltage (+15 kV) and large negative voltage (−10 kV) were applied to the needle and Al collector, respectively. During electrospinning, the distance between the tip of the needle and collector was fixed to 0.2 m, and the feed rate was set to 0.01 mL/h. The as-synthesized NFs were annealed at 600 °C for 2 h to remove the organic matter and water to obtain crystalline phases.

### 2.3. Functionalization of Pd Nanoparticles

Pd NPs were deposited on the surface of In_2_O_3_-loaded ZnO NFs using a UV reduction method. A Pd^2+^-containing solution was prepared by dissolving 0.4 g PdCl_2_ (Kojima chemicals Co. Ltd., Saitama, Japan) in 20 g of isopropanol. The In_2_O_3_-loaded ZnO NFs were immersed completely in the Pd^2+^-containing solution. Subsequently, the solution was irradiated with UV light at 360 nm using a halogen lamp at room temperature. The intensity of UV light was fixed to 0.17 mW/cm^2^ for 60 s. After the UV exposure, the NFs were removed from the solution and annealed at 500 °C for 1 h to remove any residual solvent.

### 2.4. Materials Characterization

The morphology of the products was observed by field-emission scanning electron microscopy (FE-SEM, Hitachi, S-4200, Tokyo, Japan) and transmission electron microscopy (JEM-3010, Tokyo, Japan). The elemental composition was determined by energy-dispersive X-ray spectroscopy (EDS). X-ray diffraction (XRD) using Cu Kα radiation (λ = 0.15094 nm) was used to examine the crystallinity and phase formation. Ultraviolet photoelectron spectroscopy (UPS, Thermo Fisher Scientific Co., K-Alpha+, Waltham, USA) was carried out in an ultrahigh vacuum (UHV) chamber through the irradiation of HeI 21.2 eV light to construct the electronic band structures of the components by obtaining the work functions.

### 2.5. Gas Sensing Measurement

The sensing measurements performed in this study are explained in detail elsewhere [35,36]. Here, only the basic points are described. First, Ti (50 nm thick) and Pt (100 nm thick) bilayer electrodes were sputter-deposited onto a SiO_2_-grown Si substrate and NFs were then drop-casted onto the substrate with a solvent of 2-propanol. For pure Pd sensors, Pd NPs were synthesized on Si wafers by adopting the same experimental conditions used for the functionalization of Pd NPs. The synthesized Pd NPs were drop-casted on the same Si substrate equipped with electrodes and the gas sensing properties were studied.

Second, for the sensing measurements, the fabricated sensor devices were placed into a tube furnace, equipped with a gas chamber that can control the temperature. The gas concentrations were controlled by varying the ratios of the desired gases to dry air using mass flow controllers. After recording the resistance in air (*R_a_*) and the resistance in the presence of the target gas (*R_g_*) using a Keithley source meter, the sensor response (*R*) was calculated as *R = R_a_*/*R_g_* (for a reducing gas, such as H_2_ and CO) and *R = R_g_*/*R_a_* (for an oxidizing gas, such as NO_2_). The response and recovery times were defined as the time required for the resistance to reach 90 % of its final value upon exposure to target gas and air, respectively [37].

## 3. Results and Discussion

### 3.1. Morphological and Compositional Studies

**Figure 1:**
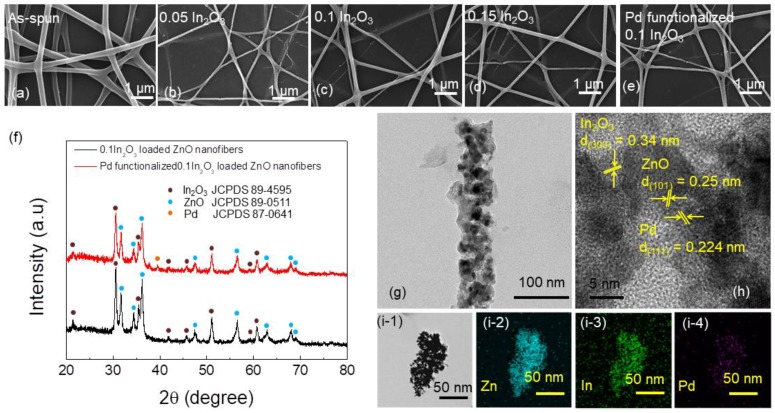
Field emission scanning electron microscopy (FE-SEM) images of In_2_O_3_-loaded ZnO nanofibers (NFs) with different amounts of In_2_O_3_ (**a**) as-spun In_2_O_3_-loaded ZnO NFs. (**b**) Calcined 0.05 In_2_O_3_-loaded ZnO NFs, (**c**) calcined 0.1 In_2_O_3_-loaded ZnO NFs (**d**) calcined 0.15 In_2_O_3_-loaded ZnO NFs. (**e**) FE-SEM images of calcined Pd nanoparticle (NP)-functionalized 0.1 In_2_O_3_-loaded ZnO NFs (**f**) XRD patterns of 0.1 In_2_O_3_-loaded ZnO NFs and Pd-functionalized 0.1 In_2_O_3_-loaded ZnO nanofibers. (**g**) A typical TEM image of 0.1 In_2_O_3_-loaded ZnO NFs. (**h**) High-resolution TEM (HRTEM) image of Pd NP-functionalized 0.1 In_2_O_3_-loaded ZnO NFs. (**i-1**)–(**i-4**) Energy-dispersive X-ray spectroscopy (EDS) elemental mapping analysis of Pd NP-functionalized 0.1 In_2_O_3_-loaded ZnO NFs.

Figure 1a presents a representative FE-SEM image of as-spun In_2_O_3_ (0.1 molar ratio)-loaded ZnO NFs prior to calcination; it shows a smooth morphology with a web-like structure. Figure 1b–d shows FE-SEM images of ZnO NFs loaded with 0.05, 0.1, and 0.15 In_2_O_3_, respectively, after calcination. The insets in these figures show higher-magnification FE-SEM images. Overall, the diameter of the synthesized NFs was less than 100 nm and the smooth morphology observed on the as-spun NFs was changed to an irregular and rough morphology, even though the overall web-like structure of NFs had been maintained. The rough morphology was attributed to the evaporation and decomposition of water and inorganic materials during the calcination process.

Figure 1e presents FE-SEM images of Pd NP-functionalized 0.1 In_2_O_3_-loaded ZnO NFs. Similar to the pristine In_2_O_3_-loaded ZnO NFs, the surface morphology became rough. In addition, the overall diameter was less than 100 nm, demonstrating a nanoscale range of synthesized NFs. Figure 1f shows XRD patterns of 0.1 In_2_O_3_-loaded ZnO nanofibers and Pd-functionalized 0.1 In_2_O_3_-loaded ZnO nanofibers. The XRD pattern of the 0.1 In_2_O_3_-loaded ZnO nanofibers revealed crystalline In_2_O_3_ (JCPDS(joint committee on powder diffraction standards) card no. 89-4595 [38]) and crystalline ZnO (JCPDS card no. 89-0511 [39]). For Pd-functionalized 0.1 In_2_O_3_-loaded ZnO nanofibers an additional peak related to Pd (JCPDS card no. 87-0641 [40]) appears. This demonstrates the formation and co-existence of crystalline Pd, In_2_O_3_, and ZnO phases. In addition, no unwanted phases were observed, demonstrating the high purity of the starting materials and the correctness of the procedure employed for the preparation of NFs.

**Figure 2:**
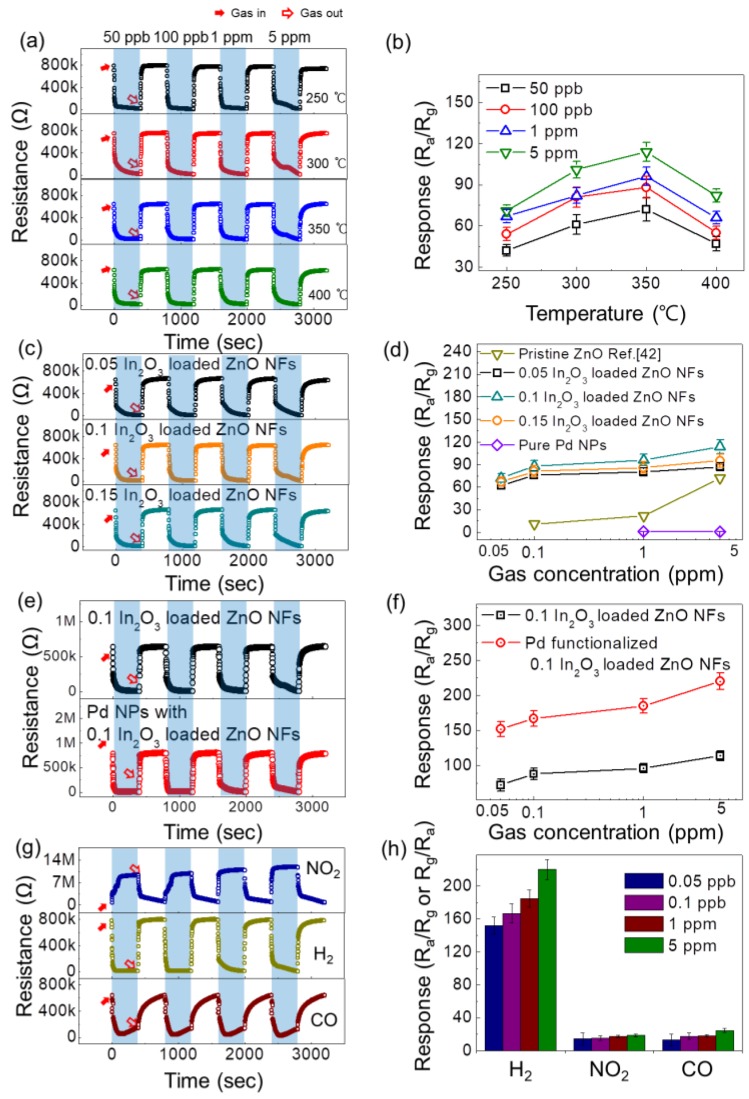
(**a**) Dynamic resistance curves of the 0.1 In_2_O_3_-loaded ZnO NFs gas sensor to various concentrations of hydrogen gas at different temperatures. (**b**) Response versus sensing temperature. (**c**) Dynamic resistance curves of the In_2_O_3_-loaded ZnO NFs with different In_2_O_3_ loadings to various concentrations of hydrogen gas at 350 °C. (**d**) Calibration curves of pristine ZnO [42], pure Pd NPs (at 200 °C), and In_2_O_3_-loaded ZnO NFs with different In_2_O_3_ contents. (**e**) Dynamic resistance curves of 0.1 In_2_O_3_-loaded ZnO NFs and Pd NP-functionalized 0.1 In_2_O_3_-loaded ZnO NFs gas sensors to different concentrations of H_2_ at 350 °C. (**f**) Response versus H_2_ gas concentration. (**g**) Dynamic resistance curves of Pd NP-functionalized 0.1 In_2_O_3_-loaded ZnO NFs gas sensors to different concentrations of NO_2_, H_2_, and CO gases at 350 °C. (**h**) Selectivity histogram of Pd NP-functionalized 0.1 In_2_O_3_-loaded ZnO NFs gas sensor.

**Figure 3:**
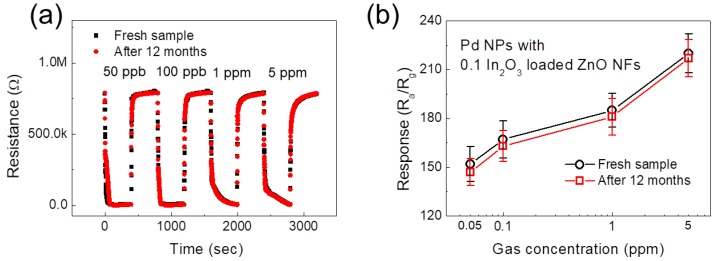
(**a**) Dynamic resistance curves of the fresh and 12-months-aged Pd NP-functionalized 0.1 In_2_O_3_-loaded ZnO NFs gas sensor. (**b**) Corresponding calibration curves.

**Figure 4:**
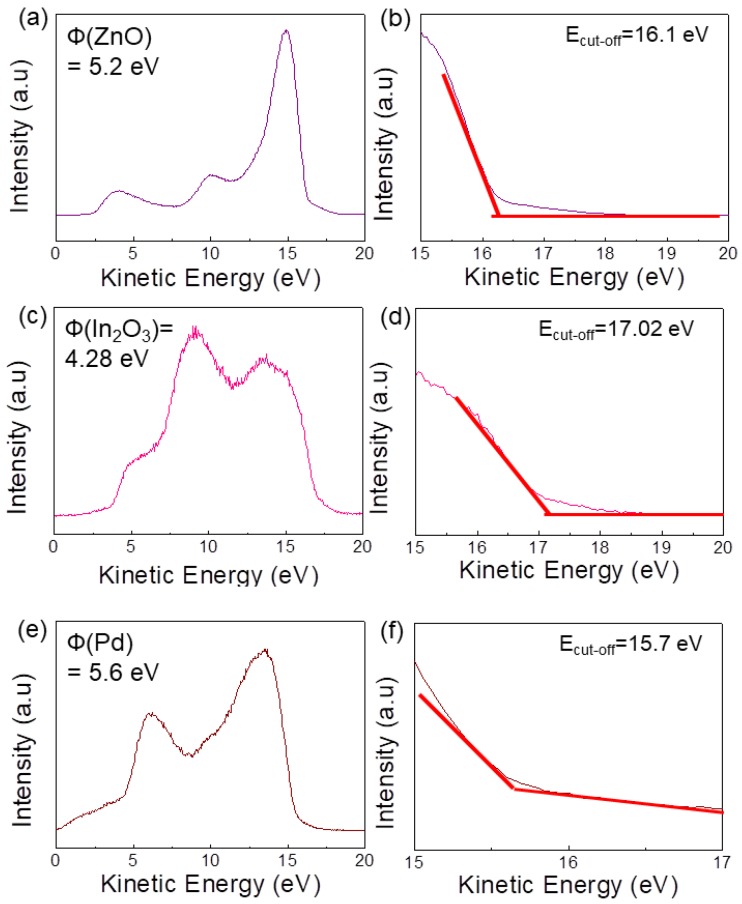
(**a**,**c**,**e**) ultraviolet photoelectron spectroscopy (UPS) spectra of ZnO, In_2_O_3_, and Pd. (**b**,**d**,**f**) cutoff values of UPS spectra for ZnO, In_2_O_3_, and Pd, respectively.

TEM of the Pd-functionalized 0.1 In_2_O_3_-loaded ZnO NFs was performed to achieve better insight into the microstructure. Figure 1g shows a typical TEM image of Pd-functionalized 0.1 In_2_O_3_-loaded ZnO nanofibers, clearly showing the presence of nanograins on the surface of the products. The corresponding high-resolution TEM (HRTEM) image (Figure 1h) shows fringes with a spacing of 0.34, 0.25, and 0.224 nm, which can be assigned to the (300), (101) [41], and (111) crystalline planes of In_2_O_3_, ZnO, and Pd, respectively. Figure 1i-1–i-4 show EDS elemental mapping analysis of Pd-functionalized 0.1 In_2_O_3_-loaded ZnO nanofibers. This shows the spatial distribution of Zn, In, and Pd elements on the synthesized NFs.

### 3.2. Gas Sensing Studies

In the first step, an attempt was made to find the optimal sensing temperature using the 0.1 In_2_O_3_-loaded ZnO NF gas sensor while exposing it to low concentrations of hydrogen gas (50 ppb–5 ppm) at a range of sensing temperatures. Figure 2a shows the transient resistance curves at 250–400 °C. At all temperatures tested, the sensor exhibited very high reproducibility of the resistance signal, where after stopping hydrogen gas, the resistance returned to its initial value. In addition, the sensor exhibited n-type sensing behavior, originating from the n-type conductive nature of both ZnO and In_2_O_3_. To examine the temperature dependence of the gas response, the response of the gas sensor at various hydrogen concentrations versus the sensing temperature was plotted, as shown in Figure 2b. From this plot, the optimal sensing temperature was determined to be 350 °C. At lower temperatures, the thermal energy is insufficient to provide the complete energy of adsorption, and at 400 °C hydrogen gas may escape before adsorption. At the optimal sensing temperature, the adsorption and desorption rates are equal. The responses to 50 ppb, 100 ppb, 1 ppm, and 5 ppm H_2_ gas were 72, 88, 96, and 114, respectively. This demonstrates the strong response of the gas sensor to hydrogen gas.

In the next step, the gas sensors with various amounts of In_2_O_3_ were exposed to hydrogen gas at 350 °C to determine the optimal composition of the gas sensor. Figure 2c,d shows the transient resistance curves of different sensors and corresponding calibration curves, respectively. Note that the data for the pristine ZnO NF gas sensor were taken from reference [42]. Based on the presented results, the 0.1 In_2_O_3_-loaded ZnO NF gas sensor showed the strongest response to all concentrations of H_2_ gas. The responses of the ZnO nanofibers loaded with 0.05, 0.1, and 0.15 In_2_O_3_ toward 50 ppb H_2_ gas were 62, 72, and 67 respectively, again clarifying the trend.

It should be noted that we also studied the sensing results for the pure Pd sensor. As shown in Figure 2d, the sensing response of the pure Pd sensor is inferior to the other sensors. Figure S1a,b in Supplementary Materials shows the dynamic resistance curves and calibration curves of the pure Pd gas sensor to H_2_ gas at different operating temperatures, respectively. At 200 °C, the best sensing performance was obtained and compared to the other type gases in Figure 2d.

In the next step, Pd NP-functionalized 0.1 In_2_O_3_-loaded ZnO NFs were exposed to various concentrations of H_2_ gas. Figure 2e shows the transient response curves of the 0.1 In_2_O_3_-loaded ZnO NFs and Pd-functionalized In_2_O_3_-loaded ZnO NFs. The corresponding calibration curves presented in Figure 2f shows that the gas sensor functionalized with Pd NPs showed a much improved response to H_2_ gas compared to the In_2_O_3_-loaded ZnO NFs without Pt functionalization. Accordingly, the Pd-functionalized 0.1 In_2_O_3_-loaded ZnO NF gas sensor was selected for the selectivity study.

Figure 2g shows the dynamic resistance curves of the Pd-functionalized 0.1 In_2_O_3_-loaded ZnO MF gas sensor to 50 ppb–5 ppm NO_2_, CO, and H_2_ gases. Figure 2h shows the corresponding selectivity pattern, demonstrating the excellent selectivity of gas sensor to hydrogen gas. Considering 50 ppb gases, the responses of the gas sensor to H_2_, NO_2_, and CO gases were 152, 14.6, and 13.48, respectively. Long-term stability of sensors was also tested. Figure 3a,b shows the dynamic resistance curves and corresponding calibration curves of fresh and 12-months-aged Pd NPs-functionalized 0.1 In_2_O_3_-loaded ZnO NFs gas sensors toward 50 ppb–5 ppm of H_2_ gas, respectively. As is evident, after 12 months the sensors showed almost the same sensing response, demonstrating the high stability of the gas sensor which is critical for practical applications. Long-term stabilities of the other gas sensors are shown in Figure S2 in Supplementary Materials.

### 3.3. Gas Sensing Mechanism

When chemiresistor-based gas sensors are exposed to different gases, their resistance changes, which is the basis of gas detection in these types of gas sensors. Any resistance modulation source can contribute to the sensor signal. Let us start with the electron depletion layer (EDL) concept [43]. When chemiresistor-based gas sensors are exposed to air, oxygen gases can be adsorbed easily on the surface and take the electrons from the sensor materials. As a result, the oxygen ion species (O_2_^−^, O^−^, and O^2−^) become chemisorbed on the sensor surface. On the other hand, due to the depletion of the outer layer of the sensing material, the concentration of electrons decrease, which leads to the appearance of a so-called EDL. When the resistance of gas sensor in the air was compared with that in the vacuum, the resistance of sensor in the air was higher due to the presence of the EDL. Upon exposure of the gas sensor to a toxic gas, the toxic gas molecules will react with already adsorbed oxygen ion species and in the case of reducing gases, they will release electrons to the surface of the gas sensor. As a result, the width of the EDL decreases, leading to resistance modulation. This eventually leads to the appearance of a sensor signal. In the In_2_O_3_-loaded ZnO nanofibers in the surface of each individual grain of ZnO and In_2_O_3_, which are exposed to air, EDLs form and upon exposure to H_2_ gas, the following reaction, H_2_ + O^−^ → H_2_O + e^−^, will cause the electrons to return to the surface of the gas sensor. Accordingly, the resistance of the sensor changes. In addition, in In_2_O_3_-loaded ZnO nanofibers, heterojunctions between In_2_O_3_ and ZnO form because of the co-existence of In_2_O_3_ and ZnO and the difference in work function. The work function values were obtained according to the procedure described elsewhere [44].

Figure 4a,c,e shows the UPS spectra of ZnO, In_2_O_3_, and Pd, respectively. The cutoff values of 16.1 eV (ZnO), 17.02 eV (In_2_O_3_), and 15.70 eV (Pd) were obtained from Figure 4b,d,f. The work function can be calculated by subtracting the cutoff values from the injected photon energy. To correct the broadening of the analyzer, 0.1 eV should be added to the work function. Consequently, the work functions of ZnO, In_2_O_3_, and Pd were calculated to be 5.2 eV (i.e., 21.2–16.1 + 0.1 = 5.2 eV), 4.28 eV (i.e., 21.2–17.02 + 0.1 = 4.28 eV), and 5.6 eV (i.e., 21.2–15.7 + 0.1 = 5.60 eV), respectively.

**Figure 5:**
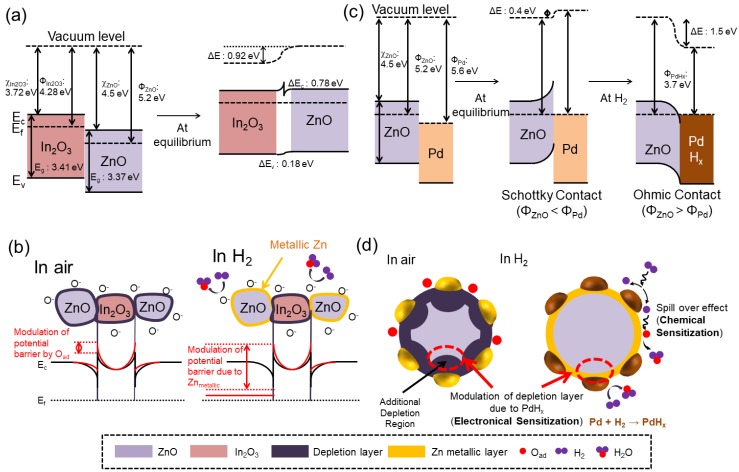
(**a**) Energy levels of In_2_O_3_ and ZnO before and after contact in a vacuum. (**b**) Energy levels of ZnO and In_2_O_3_ after contact in air and hydrogen atmospheres. (**c**) Energy levels of Pd and ZnO before and after contact in air and hydrogen atmospheres. (**d**) Electronic and chemical sensitization effects of Pd.

According to the calculated work functions, the energy levels can be established. As shown in Figure 5a, upon intimate contact between In_2_O_3_ and ZnO, and to equate the Fermi levels, electrons flow from In_2_O_3_ to ZnO and band bending occurs. This will result in a potential barrier for the flow of electrons. When the sensor is exposed to H_2_ gas, the electrons released return to the surface of the sensing layer, eventually decreasing the height of the potential barrier. This leads to a decrease in the sensor resistance, which contributes to the sensor signal. In addition, ZnO–In_2_O_3_ heterojunctions, ZnO–ZnO homojunctions, and In_2_O_3_–In_2_O_3_ homojunctions also can be formed. On the other hand, because ZnO is the major component, the contribution of ZnO–ZnO homojunctions to the sensing signal is significantly higher. At ZnO–ZnO homojunctions, the height of the potential barriers in air changes greatly in a hydrogen atmosphere, contributing predominantly to the sensor signal.

Because of the high sensing temperature and high reducing power of hydrogen gas, it is possible that zinc oxide can be converted to metallic zinc on the surface of ZnO (Figure 5b). This conversion is one of the main reasons for the enhanced sensing response because metallic Zn has a significantly higher conductivity than ZnO [33]. When ZnO is in a H_2_ atmosphere, the continuous ultra-thin layer on the outer surfaces of ZnO will be reduced to metallic Zn, where a semiconductor-to-metallic conversion will occur [42]. Because the resistance of ZnO is significantly higher than that of metallic Zn, remarkable resistance modulation occurs upon this conversion, which contributes to the sensing signal [3]. Based on the literature, [45] metallization of the ZnO surface can occur by the adsorption of H atoms onto the O sites on the nonpolar surfaces of ZnO, resulting in the metallization of ZnO. Hybridization occurs between the s-orbitals of H atoms and the p-orbitals of O atoms (in ZnO), resulting in a shift in the energy states of these O p-orbitals shift to lower energy states. Accordingly, delocalization of the charges between Zn and the O–H bond occurs, which can lead to the metallization of Zn atoms.

In addition, the presence of Pd can increase the H_2_ response of the gas sensor significantly. Pd can dissociate oxygen molecules to atomic oxygen and increase the rate of oxygen adsorption on the surface of the gas sensor [4]. According to the UPS measurements, the work function of Pd was measured to be 5.60 eV. Therefore, Schottky barriers are formed at the interface between Pd and ZnO due to electron transfer from ZnO to Pd. This causes an overall increase in the resistance of the gas sensor. On the other hand, upon exposure to H_2_ gas, it can be dissociated on and dissolved into the Pd. Pd can uptake more than 600 times its own volume of H_2_ gas [46]. Accordingly, Pd can adsorb H_2_ and form PdH_x_. The formation of PdH_x_ with a very different work function (3.7 eV) than Pd will destroy the Schottky barriers to form Ohmic contact (Figure 5c) [33], which can eventually modulate the sensor resistance. The formation of PdH_x_ can be shown as follows [46]:

H_2_ → 2H, (1)

Pd + xH → PdH_x_. (2)

At the same time, partial H atoms formed dissociatively in Pd migrate to the interface of Pd and the sensing layer via a so-called spill-over effect (Figure 5d). The formed PdH_x_ and migrated H atoms potentially react with the adsorbed oxygen and inject electrons into the sensing layer, resulting in a decrease in resistance. The reduced concentration of H atoms at the interface of Pd and the sensing layer may assist in the dissolution of H to Pd, which certainly promotes the sensor performance. The following chemical formulae are a possible explanation of the complete reaction [47].

(3) $${\mathrm{PdH}}_{x}+\frac{x}{2}O^{-}\;\left(\mathrm{ads}\right)\to \mathrm{Pd}+\frac{x}{2}H_{2}O+\frac{x}{2}e^{-},$$

O^−^ (ads) + 2H → H_2_O + e^−^. (4)

Another possible mechanism can be related to the kinetics of gas molecules. The strong response to H_2_ gas can also be related to the smaller kinetic diameter of H_2_ gas (2.89 Å) [48] relative to CO (3.76 Å) and NO_2_ gas (4.01–5.02 Å) [49]. Accordingly, H_2_ gas molecules can diffuse to the deeper parts of the sensing layer. Therefore, their adsorption volume can be significantly larger than that of other gases.

Table 1 lists the H_2_ sensing properties of the present sensor with some ZnO-based gas sensors reported elsewhere. Overall, the present sensor showed a superior response to H_2_ gas, demonstrating its practical applications.

## 4. Conclusions

Pd-functionalized xIn_2_O_3_ (x = 0.05, 0.1, and 0.15)-loaded ZnO NFs were prepared for H_2_ gas sensing applications. FE-SEM, TEM, and XRD were performed to confirm the desired morphology and chemical composition of the products. The gas sensing results showed that the optimal gas sensor with Pd-functionalized 0.1 In_2_O_3_-loaded ZnO NFs showed a very strong response to 152–50 ppb H_2_ gas at 350 °C. The enhanced gas response and excellent selectivity were attributed to the excellent catalytic activity of Pd to H_2_ gas, formation of PdH_x_, formation of Pd/ZnO Schottky junctions, formation of In_2_O_3_/ZnO heterojunctions, as well as ZnO–ZnO homojunctions. The optimized gas sensor has almost all the features of a good sensor for practical applications. Moreover, based on the results, further enhancement in the gas response may be achieved by optimizing the content of Pd NPs.

## Figures and Tables

**Table 1:** Comparison of the hydrogen sensing performances of some ZnO-based gas sensors with the present optimized gas sensor.

Sensing Material	Conc. (ppm)	T (°C)	Response (*R_a_*/*R_g_*)	Ref.
Pd-functionalized 0.1 In_2_O_3_-loaded ZnO NFs	50 ppb	350	172	Present work
150 kGy-irradiated ZnO NFs	10	350	150	[3]
ZnO–SnO_2_ composite	10,000	150	10	[11]
Au-functionalized SnO_2_–ZnO NWs (nanowires)	0.1	300	8.9	[50]
Pd-functionalized ZnO NWs	100	350	87.17	[33]
Pd-functionalized ZnO nanoflowers	300	300	2.8	[51]
WO_3_-decorated ZnO NWs	5000	200	12.6	[52]
Pt/Pd-decorated ZnO nanorods	10,000	100	69.8	[53]

## Supplementary Materials

The following are available online at https://www.mdpi.com/1424-8220/19/19/4276/s1, Figure S1: (a) Dynamic resistance curves of the pure Pd gas sensor to various concentrations of H_2_ gases at different temperatures. (b) Corresponding calibration curves. Figure S2: Dynamic resistance curves and corresponding response versus H_2_ gas concentration of the fresh and 12 months aged (a) and (b) 0.05, (c) and (d) 0.1 and (e) and (f) 0.15 In_2_O_3_-loaded ZnO NFs gas sensors, respectively.
