# Supplementary material for: ppb-Level Selective Hydrogen Gas Detection of Pd-Functionalized In2O3-Loaded ZnO Nanofiber Gas Sensors
Source: Sensors (Basel). 2019 Oct 2;19(19):4276. doi: 10.3390/s19194276 (PMC6806323; doi:10.3390/s19194276)
Supplement: Supplementary file 1 [file sensors-19-04276-s001.pdf]

Supplementary material

ppb-Level selective hydrogen gas detection of Pd-functionalized In_2O_3-loaded ZnO nanofiber gas sensors

Jae-Hyoung Lee[1], Jae-Hun Kim[1], Jin-Young Kim[1], Ali Mirzaei [2,3], Hyoun Woo Kim[2,4], Sang Sub Kim[1,*]

1 Department of Materials Science and Engineering, Inha University, Incheon 22212, Republic of Korea; 22171057@inha.edu (J.-H.L.); kjhhb5331@hanyang.ac.kr (J.-H.K.); 22171286@inha.edu (J.-Y.K.)

2 The Research Institute of Industrial Science, Hanyang University, Seoul 04763, Republic of Korea; alisonmirzaee@yahoo.com (A. M.)

3 Department of Materials Science and Engineering, Shiraz University of Technology, Shiraz, Iran; alisonmirzaee@yahoo.com (A. M.)

4 Division of Materials Science and Engineering, Hanyang University, Seoul 04763, Republic of Korea; hyounwoo@hanyang.ac.kr (H.W.K.)

* Correspondence: sangsub@inha.ac.kr; Tel.: +82-32-860-7546 (S.S.K.)

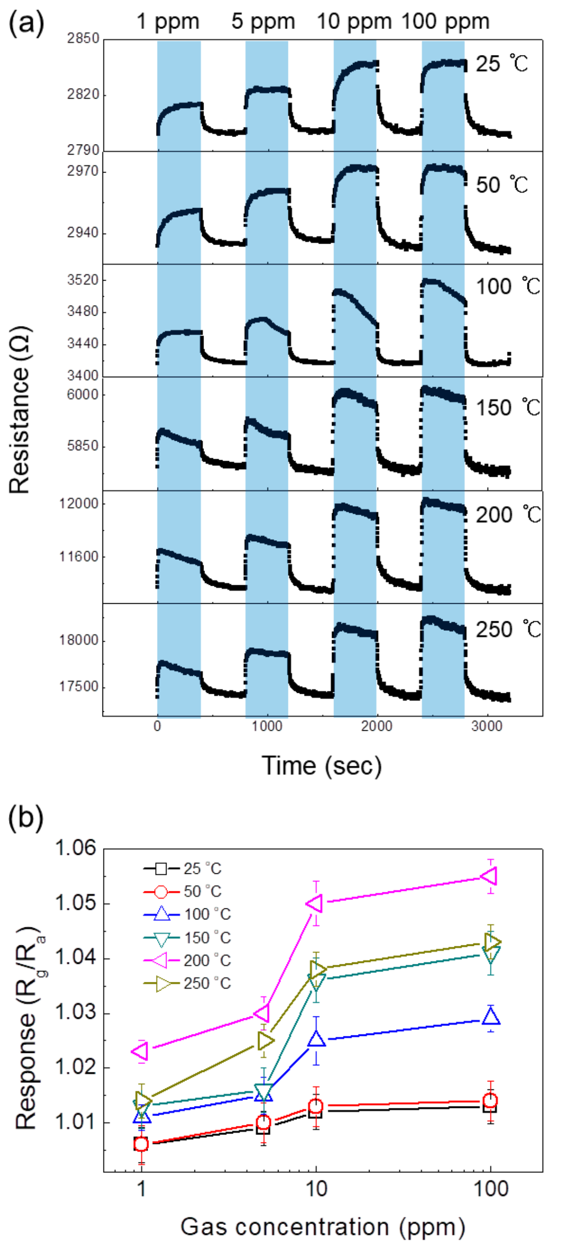

Figure S1. (a) Dynamic resistance curves of the pure Pd gas sensor to various concentrations of H_2 gases at different temperatures. (b) Corresponding calibration curves.

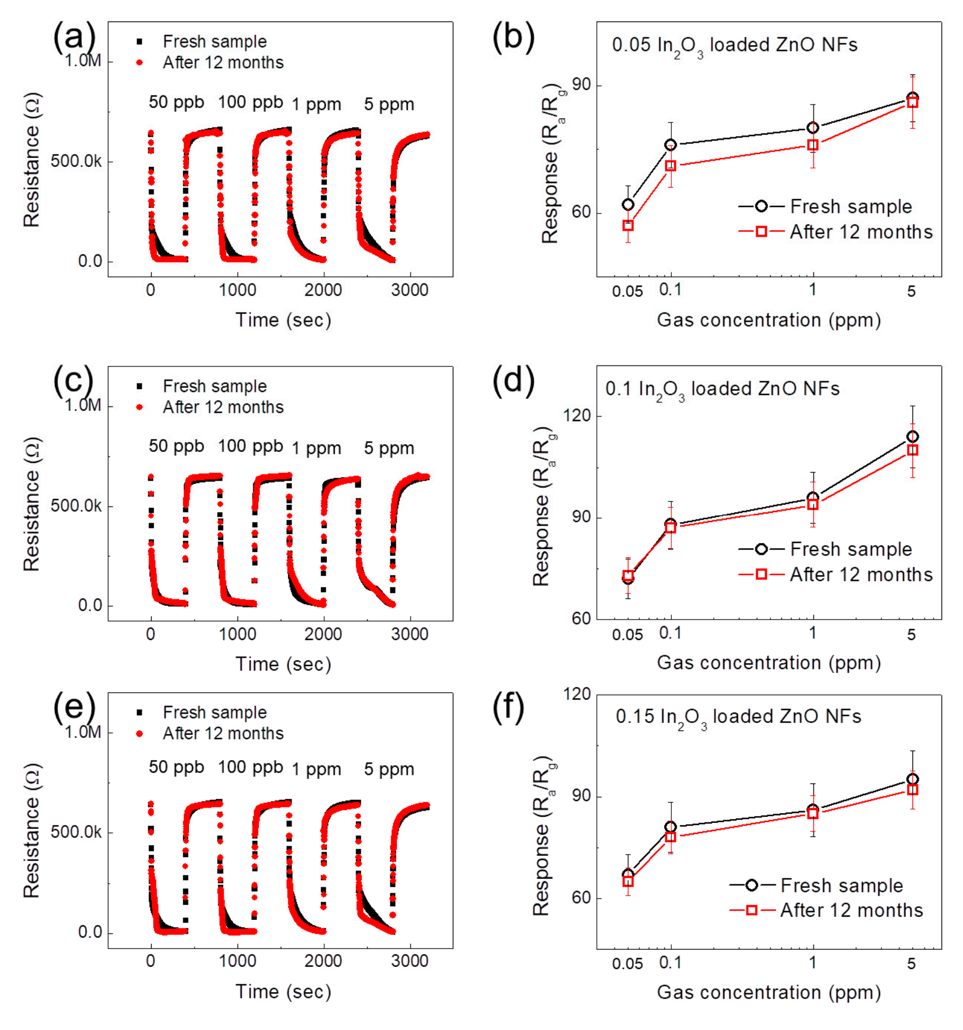

Figure S2. Dynamic resistance curves and corresponding response versus H_2 gas concentration of the fresh and 12 months aged (a) and (b) 0.05, (c) and (d) 0.1 and (e) and (f) 0.15 In_2O_3-loaded ZnO NFs gas sensors, respectively.
